# Supplementary material for: Challenges in teacher-student communication during family medicine residency: A qualitative study
Source: PLoS One. 2024 Sep 17;19(9):e0310455. doi: 10.1371/journal.pone.0310455 (PMC11407679; doi:10.1371/journal.pone.0310455)
Supplement: Attachment — Submitted filename: Response to reviewers.docx [file pone.0310455.s001.docx]

Comments to the Author

Reviewer #1: Thank you for reviewing all recommendations/suggestions and incorporating most of them. I also appreciate the clarifications where you did not incorporate suggestions - such as the response on why you felt using assertiveness was appropriate.

The overall manuscript looks in excellent shape now and is close to publication. I am submitting a recommendation to accept the manuscript rather than a minor revision, so that it saves you time and another revision is not required. However, there were two comments that were not addressed, and the response letter stated that these changes have been incorporated.

The first one is a grammatical improvement: https://www.merriam-webster.com/grammar/what-happens-to-names-when-we-make-them-plural-or-possessive

The second one is just a change of phrasing to make it more palatable.

You are welcome to incorporate these changes or retain the original, but I would recommend making sure that the grammar sounds right (in the first one).

Other than that, great work and best of luck for getting this important work published soon!

__________

Copied below from response letter - I did not find these changes in the revised version/track changes.

- Medical residency is pivotal in “specialists” training – the phrase should be specialists’ training (apostrophe after s)

- “often sidelining the development of complementary skills” – can be better phrased as “often deprioritizing’. Sideline means there is absolutely no attention to it, deprioritize indicates that the amount of attention paid to it is reduced.

Dear reviewer

Thank you very much for your positive feedback and for recommending our manuscript for acceptance. We greatly appreciate your thorough review and the valuable suggestions you have provided throughout this process.

Regarding the two comments you mentioned that were not addressed in our previous revision:

Grammatical improvement: We sincerely apologize for not incorporating the correction earlier. We have now adjusted the phrase “Medical residency is pivotal in specialists’ training” by correctly placing the apostrophe after the "s" in "specialists’" to ensure grammatical accuracy.

Change of phrasing: We also regret that the phrasing change was not included in the previous version. We have now revised the sentence “often sidelining the development of complementary skills” to “often deprioritizing the development of complementary skills,” as per your recommendation, to better convey the intended meaning.

We have made these changes in the revised manuscript, and we believe that they further improve the clarity and quality of the work.

Thank you once again for your time and feedback.
